# α-Hederin promotes ferroptosis and reverses cisplatin chemoresistance in non-small cell lung cancer

**DOI:** 10.18632/aging.205408

**Published:** 2024-01-18

**Authors:** Shugao Han, Xi Yang, Jing Zhuang, Qing Zhou, Jingjing Wang, Lixin Ru, Furong Niu, Wei Mao

**Affiliations:** 1 Department of Radiology, The Second Affiliated Hospital of Zhejiang University School of Medicine, Hangzhou 310009, China; 2 Huzhou Central Hospital, Affiliated Central Hospital Huzhou University, Huzhou 313000, China; 3 School of Medicine, Huzhou Normal University, Huzhou 313000, China; 4 Huzhou Hospital of Traditional Chinese Medicine, Zhejiang University of Traditional Chinese Medicine, Huzhou 313000, China

**Keywords:** α-Hederin, NSCLC, cisplatin-resistance, ferroptosis, DDIT3

## Abstract

Background: Cisplatin is a core chemotherapy regimen in non-small cell lung cancer (NSCLC). However, chemoresistance to cisplatin leads to a poor prognosis in NSCLC. α-Hederin is a natural compound extracted from *Nigella sativa*. The study aims to explore the effects of α-Hederin on cisplatin resistance in NSCLC.

Methods: NSCLC cisplatin-resistant cell lines A549/DPP and PC-9 were cultured to evaluate the efficacy of α-Hederin in the treatment of NSCLC *in vitro* and *in vivo*. Metabolomics and RNA-seq analysis were used to determine the potential mechanisms of action of α-Hederin.

Results: The results showed that α-Hederin inhibited cisplatin-resistant NSCLC cells proliferation and metastasis. Mice xenograft, orthotopic, and metastatic A549/DPP cell models also showed the anti-tumor effects of α-Hederin. The metabolomics and RNA-seq analysis results showed that α-Hederin activated DDIT3/ATF3 pathway and ferroptosis via silencing SLC7A11 and GPX4. Furthermore, α-Hederin enhanced the nuclear expression of EGR1. Bioinformatics and luciferase experiments confirmed that EGR1 binds to the miR-96-5p promoter region, inhibiting transcription. In addition, miR-96-5p directly suppressed the levels of DDIT3.

Conclusion: This study revealed that α-Hederin activated EGR1 nuclear translocation and directly repressed miR-96-5p. It also promoted DDIT3/ATF3-mediated ferroptosis and reversed cisplatin resistance in NSCLC.

## INTRODUCTION

Lung cancer is the most common histological subtype of non-small cell lung cancer (NSCLC), accounting for over 80% of all lung cancer cases [1]. NSCLC is first diagnosed at an advanced stage in China due to the lack of widespread screening and limited diagnostic tools in most areas [2]. As a result, advanced NSCLC has a poor prognosis, with a 5-year overall survival rate as low as 15% [3]. Classification of NSCLC considers mutations at the molecular level that may affect treatment. However, most NSCLC patients (close to 85–90%) do not have an identified drug-targeted driver mutations, and thus chemotherapy remains the standard of care [4]. Surgical treatment is not preferred in advanced NSCLC. Some conventional chemotherapeutic agents are also often used after targeted drug therapy. Cisplatin (Cis/DDP), mitomycin C (MMC), paclitaxel (PTX), and doxorubicin (DOX) are commonly used as conventional chemotherapeutic agents. Cisplatin is often used clinically in a small number of patients with drug-targeted driver mutations in NSCLC. The main drawback of conventional chemotherapy is the limited tumor suppression and toxic side effects, including nausea, vomiting, hair loss, fatigue, and diarrhea. In addition, residual tumor cells and systemic toxicity associated with chemotherapy can exacerbate host immunosuppression, thus increase tumor progression [5, 6]. Effective chemotherapy improves the quality of life and overall survival rate in NSCLC. The development of chemoresistance during treatment greatly limits chemotherapy efficacy and clinical utility [7]. Therefore, the resistance mechanisms in platinum-based therapies in NSCLC should be explored. In addition, appropriate measures should be taken to reduce the development of resistance in NSCLC and increase the efficacy of first-line chemotherapy.

Ferroptosis is a form of cell death distinct from apoptosis, necrosis, and autophagy. It is characterized by iron-dependent lipid peroxidation. The mitochondrial morphology is characterized by consolidation, loss of cristae, and an increase in bilayer density. Under normal physiological conditions, iron is involved in oxygen transport, DNA biosynthesis, and coenzymes in the tricarboxylic acid cycle and electron transfer. Excess intracellular iron ions catalyze the production of large amounts of reactive oxygen species (ROS), leading to cellular damage or death and inhibiting tumor cell proliferation [8, 9]. Current research suggests that ferroptosis is induced by the following: (1) elevation of intracellular divalent ferrous ions: extracellular trivalent ferric ions are bound to transferrin and then reduced to divalent ferrous ions via transferrin receptor on the cell membrane. Divalent ferrous ions are stored in a bound form to ferritin (FT). The excess divalent iron ions are oxidized to trivalent iron for extracellular transport, and this process requires Ferroprotein. An increase in transferrin receptor (TFR) and a decrease in FT and FTP associated with the transport and binding of iron, respectively, will lead to an increase in divalent iron ions inducing ferroptosis. (2) Excessive production of lipid peroxides: Through the Fenton reaction, divalent iron ions can produce precursors of lipid peroxides with hydrogen peroxide. (3) Inhibition of lipid peroxidation damage repair: Glutathione peroxidase 4 (GPX4) and xCT (reverse cystine/glutamate transporter) play an important role in this process. The xCT transporter transports extracellular cystine into the cell for the synthesis of glutathione (GSH). GPX4 uses the reduced glutathione to detoxify lipid peroxides to the corresponding alcohols or water [10]. Studies on the mechanism of cisplatin resistance have shown a high expression of GSH in the resistant cells. Ferroptosis can be induced by inhibition of GSH. Thus, ferroptosis is an area of interest in the study of cisplatin resistance. It has been shown that cisplatin resistance in head and neck cancer cells is associated with increased xCT and GPX4 [11]. In contrast, ferroptosis and cisplatin resistance in NSCLC has not been extensively studied, and the mechanism of action is unclear.

*Nigella sativa* is an herb that has been used in folk medicine for the treatment of various conditions [12–14]. It possesses anti-inflammatory, hypoglycemic, hypotensive, antioxidant, and antitumor effects [15]. α-Hederin (α-Hed) is one of its main extracts. Various studies have reported that α-Hederin possesses anti-inflammatory activity [16], relaxes airway and gastrointestinal smooth muscle [16, 17], and increases cell permeability [18]. However, studies have not reported any significant adverse effects or side effects associated with α-Hederin. The potential antitumor effects of α-Hederin have been demonstrated in breast cancer, laryngeal cancer, lung cancer, melanoma, and leukemia [19–21]. Studies have shown that α-Hederin can block the JAK2/STAT3 signaling pathway. Moreover, α-Hederin was shown to inhibit the proliferation, invasion, and migration of colon cancer cells SW620 by regulating the JAK2/STAT3 signaling pathway. α-Hederin was also shown to block the expression of JAK2 or activated STAT3, significantly inhibiting IL-6-induced epithelial-mesenchymal transition in colon cancer cells [22]. Another study also reported that α-Hederin inhibited tumor cell proliferation and induced tumor cell apoptosis-related to an imbalance of antioxidants in the cell [23]. In contrast, excessive ROS has been reported to cause downstream signaling, decrease mitochondrial Apaf-1 and Cyt C expression, increase caspase-3 and caspase-9 activity, thus activating oxidative damage of mitochondrial membrane and triggering apoptosis in breast cancer cells. Wang et al. found that α-Hederin could activate the mitochondrial apoptotic pathway by depleting intracellular GSH, thus inducing ROS accumulation and antitumor effects in hepatocellular carcinoma [24]. However, the role of α-Hederin in cisplatin resistance in NSCLC has not been reported.

The present study aimed to explore the potential effects and underlying mechanisms of α-Hederin and ferroptosis in cisplatin resistance in NSCLC.

## METHODS

### Cell treatment

A549/DDP and PC-9/DDP cells (DDP-resistant human lung cancer cells) were purchased from MeiXuan Biological Science and Technology, Inc., (Shanghai, China). The cells were cultured in F12K medium supplemented with 100 ml/l fetal bovine serum (FBS), 100 kU/l penicillin, and 100 mg/l chloramphenicol in a cell incubator at 37°C and 5% CO_2_. The drugs α-Hederin (98%; Shanghai Yuanye Bio-Technology Co., Ltd., Shanghai, China) and cisplatin (DDP; Macklin Inc., Shanghai, China) were dissolved in dimethyl sulfoxide (DMSO) and stored at −80°C.

### Cell transfection

Two siRNAs against EGR1 and DDIT3 were synthesized by GeneChem (Shanghai, China). The expression efficiency was determined using qPCR in cells transfected with vector or siRNA. miR-96-5p inhibitor and miR-96-5p mimic were constructed by Hanbio (Shanghai, China). Cells were transfected with the appropriate amount of vector using Lipofectamine 2000 (Invitrogen, Waltham, MA, USA). The cells were then cultured for 24 hours.

### Animal studies

For orthotopic assay, a total of 50 μl cell suspension of A549/DPP (1 × 10^7^) was mixed with 50 ml of Matrigel. Subsequently, the mixture was injected into the left lung of mice through the chest wall to a depth of 3 mm. Magnetic resonance imaging (MRI) was used to exam tumor formation after one week. For one treatment cycle in a week (starting from week 1 to week 6), cisplatin (5 mg/kg, intraperitoneal injection, 1 time) and α-Hederin (40 mg/kg, oral administration, 2 times) were given. Total three treatment cycles were conducted in this experiment. Each group contained five mice.

All animals’ experiments were conducted in accordance with the recommendations of the Animal Care Committee of Huzhou Central Hospital.

### Statistical analysis

Statistical analysis was performed using R (R version 3.6.3). *T*-test was used to analyze data between two independent groups, while one-way ANOVA was used to analyze differences among various groups. *p* < 0.05 was considered statistically significant.

Detailed materials and methods are available in the Supplementary Materials and Methods.

### Availability of data and materials

The data generated or analyzed during this study are included in this article, or if absent are available from the corresponding author upon reasonable request.

## RESULTS

### α-Hederin inhibited *in vitro* and *in vivo* cisplatin resistance in NSCLC

**Figure 1:**
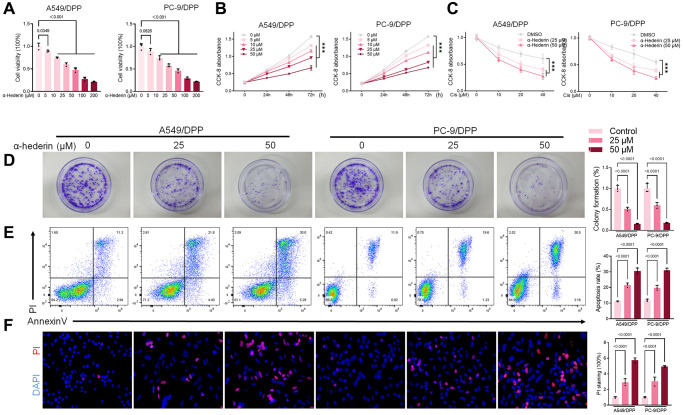
**α-Hederin reduces cisplatin resistance in cisplatin-resistant NSCLC cell lines.** (**A**) CCK8 results showing the viability of cisplatin-resistant A549/DPP and PC-9/DPP cell lines after treatment with different concentrations of α-Hederin for 24 hours. (**B**) CCK8 results showing the viability of A549/DPP and PC-9/DPP cell lines after treatment with different concentrations of α-Hederin for 0, 24, 48, and 72 hours. (**C**) CCK8 results showing the viability of cell after treatment with different concentrations of cisplatin for 24 hours. (**D**) Colony formation assay showing proliferation in NSCLC cells after treatment with α-Hederin for 24 hours. (**E**) Cell apoptosis detected by flow cytometry. (**F**) Propidium iodide (PI) staining (red) indicates apoptotic/necrotic cells. (*n* = 3). Data are shown as mean ± SD, One-way ANOVA, ^***^P < 0.001.

**Figure 2:**
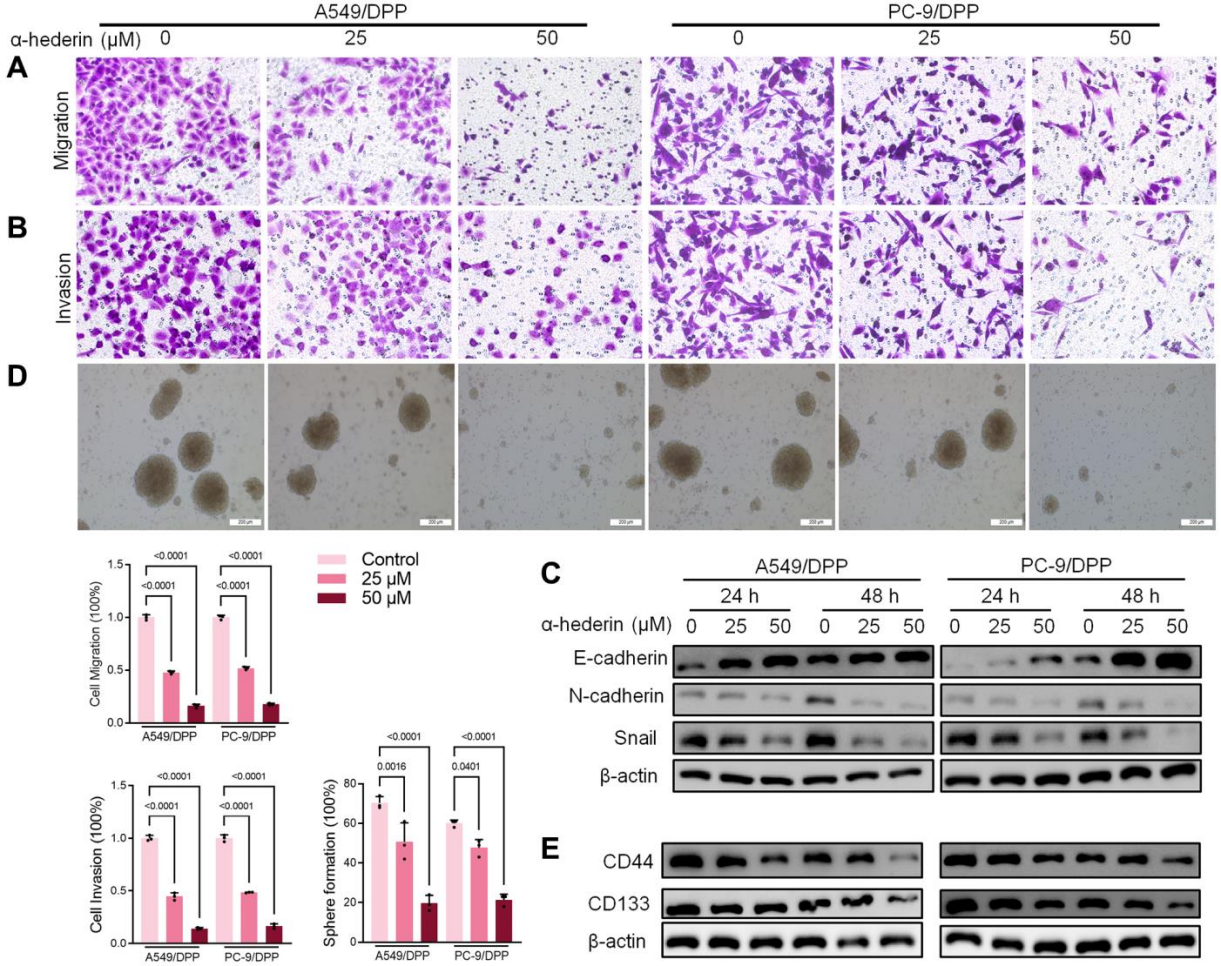
**α-Hederin decreases metastasis and stemness in cisplatin-resistant NSCLC cells.** (**A**, **B**) Cell migration and invasion were detected by Transwell assay. (**C**) Western blot demonstrating E-cadherin, N-cadherin, and Snail levels in NSCLC cells after treatment with α-Hederin for 24 hours. (**D**) The spheroid formation ability assessed in NSCLC cells. (**E**) Western blot demonstrating CD44 and CD133 levels in NSCLC cells. (*n* = 3). Data are shown as mean ± SD, One-way ANOVA.

To evaluate the effect of α-Hederin on cisplatin resistance in NSCLC, we first cultured cisplatin resistance NSCLC A549/DPP and PC-9/DPP cell lines. The cells were then treated with α-Hederin for 24 h, to suppress the viability of the cell lines in a dose-dependent manner (Figure 1A). A CCK8 assay determined at 0, 24, 48, and 72 hours showed that α-Hederin inhibited cell proliferation (Figure 1B). In addition, α-Hederin sensitized A549/DPP and PC-9/DPP cells to cisplatin in a dose-dependent manner (Figure 1C). Colony formation assay showed that A549/DPP and PC-9/DPP treated with 25 and 50 μM of α-Hederin significantly inhibited cell proliferation (Figure 1D). Cell apoptosis was determined via flow cytometry and Propidium Iodide (PI) staining after treatment with α-Hederin for one day. As shown in Figure 1E, 1F, α-Hederin significantly promoted cell apoptosis. To assess the effect of α-Hederin on cell metastatic ability, Transwell assay was used to detect cell migration and invasion, while Western blot was used to determine protein levels of E-cadherin, N-cadherin, and snail. Figure 2A–2C shows that α-Hederin abrogates cell migration and invasion. The results showed that N-cadherin and snail protein expression were suppressed while E-cadherin protein expression was enhanced. α-Hederin at a concentration of 25 and 50 μM was also shown to weaken the sphere-forming ability of NSCLC cells and decrease the protein levels of stemness markers CD133 and CD44 (Figure 2D, 2E).

**Figure 3:**
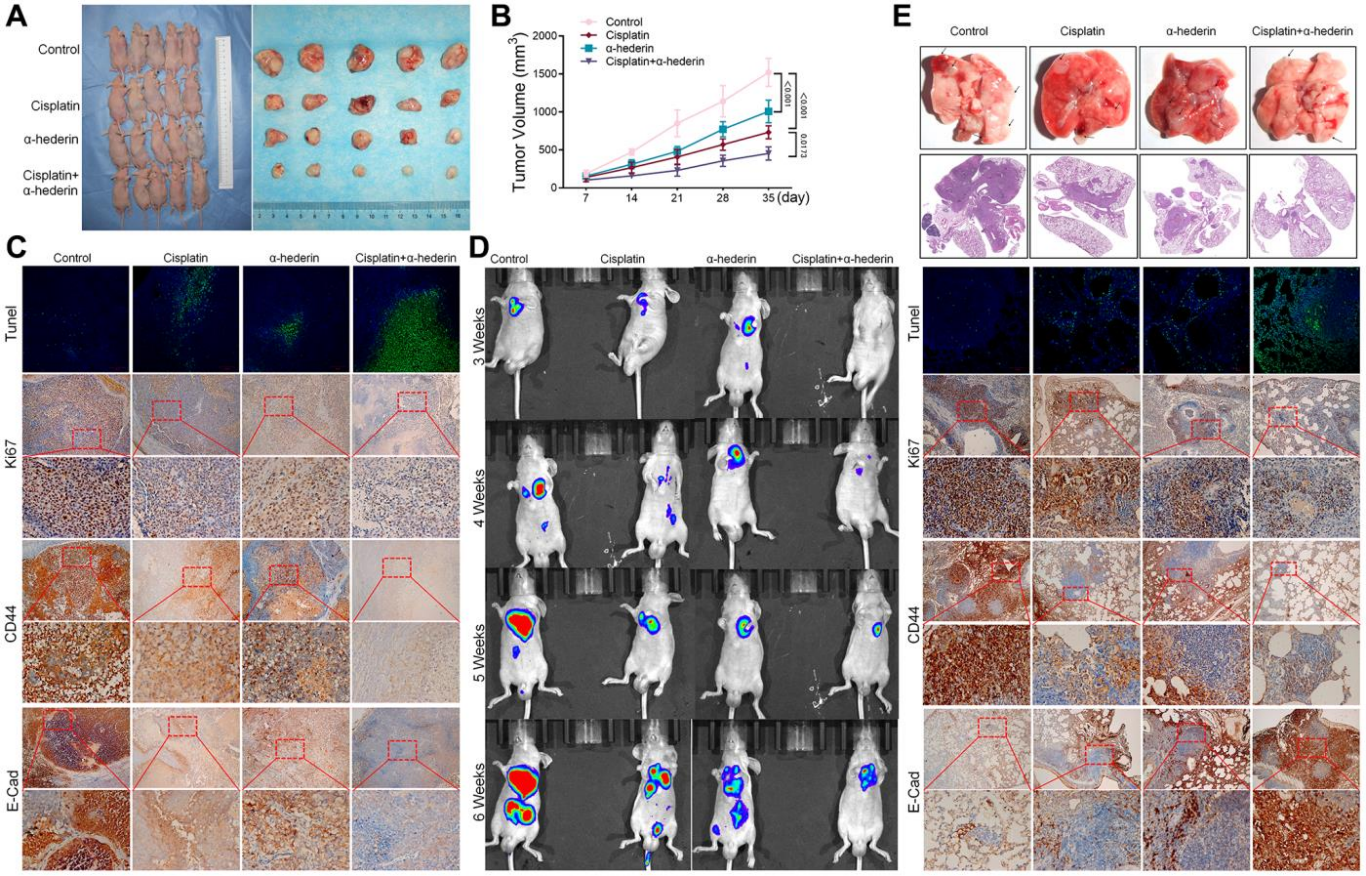
**α-Hederin decreases A549/DPP cell growth *in vivo*.** (**A**) Representative images of xenograft tumors isolated from nude mice in the different groups. (**B**) Tumor sizes in the different groups. (**C**) Representative images of Tunel, Ki-67, CD44, E-Cad staining isolated from nude mice xenograft tumor tissues in the different groups. (**D**) Live imaging showing metastasis of A549/DPP cells of an intravenous tail injection with α-Hederin in different weeks. (**E**) Representative images of orthotopic tumor model isolated from nude mice in the different groups and HE, Tunel, Ki-67, CD44, E-Cad staining in orthotopic lung tissue. (*n* = 5). Data are shown as mean ± SD, One-way ANOVA, ^*^P < 0.05, ^**^P < 0.01, ^***^P < 0.001.

The mice xenograft model of A549/DPP tumor construction confirmed α-Hederin (40 mg/kg) treatment repressed tumor volume compared with the control group. Moreover, α-Hederin (40 mg/kg) combined with cisplatin (5 mg/kg) further inhibited xenograft tumor formation compared with cisplatin treatment (Figure 3A, 3B). Immunohistochemical detection indicated that α-Hederin (40 mg/kg) treatment increased Tunel and E-cad and suppressed Ki-67 and CD44 expression in xenograft tumor tissues (Figure 3C). Live imaging showed that α-Hederin (40 mg/kg) combined with cisplatin (5 mg/kg) abrogated NSCLC metastasis compared with cisplatin treatment (Figure 3D). Orthotopic models of lung tumor tissues were also reproduced in nude mice in the different treatment groups (Figure 3E).

### α-Hederin treatment promoted ferroptosis in NSCLC cells

**Figure 4:**
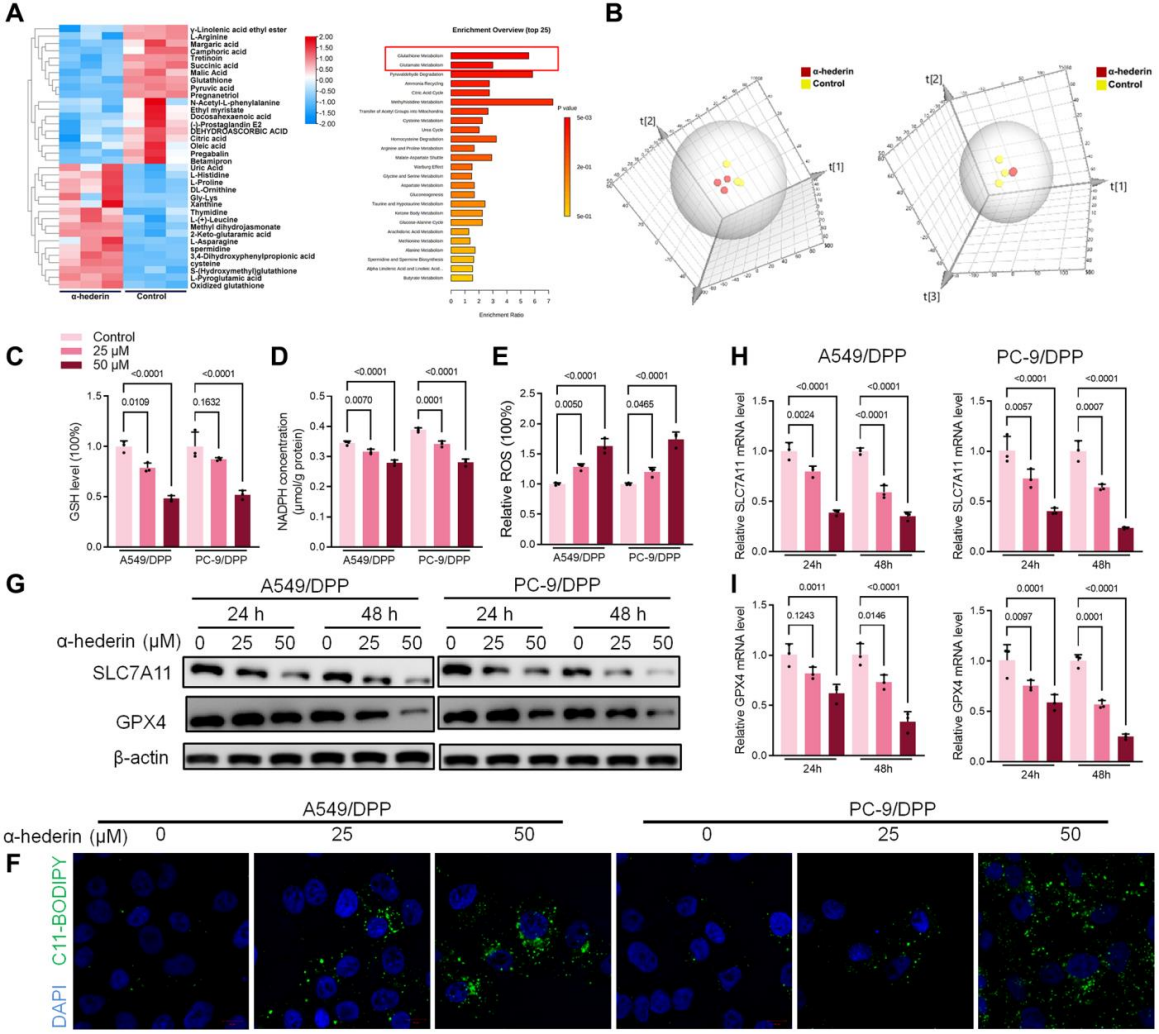
**α-Hederin treatment activated ferroptosis in NSCLC.** (**A**) OPLS-DA score plot showing differences between α-Hederin treatment and control groups in A549/DPP cells. (**B**) A heat map was used to identify metabolites in A549/DPP cells. (**C**) Cellular GSH level was detected in NSCLC cells after treatment with α-Hederin for 24 hours. (**D**) Cellular NADPH level was detected in NSCLC cells. (**E**) Cellular ROS level was detected in NSCLC cells. (**F**) Immunofluorescence assays were performed using C11-BODIPY (green) to assess oxidation and DAPI staining to visualize the Nuclei (blue) in NSCLC cells. (**G**) Western blot demonstrating SLC7A11 and GPX4 protein levels in NSCLC cells after treatment with α-Hederin for 24 hours. (**H**, **I**) qRT-PCR demonstrating SLC7A11 and GPX4 mRNA levels in NSCLC cells. (*n* = 3). Data are shown as mean ± SD, One-way ANOVA.

MetaboAnalyst software was used to explore underlying alterations in metabolic pathways. As shown in Figure 4A, 4B, α-Hederin significantly altered the glutathione pathway. GSH depletion and lipid peroxidation are significant hallmarks of ferroptosis related cell death. Thus, we evaluated ferroptosis and lipid peroxidation-related indicators such as GSH, NADPH, and ROS levels. Results showed that α-Hederin depleted cellular GSH and nicotinamide adenine dinucleotide phosphate (NADPH) concentration but stimulated the ROS levels (Figure 4C–4E). Besides, Immunofluorescence using C11-BODIPY also showed that α-Hederin induced lipid peroxidation in NSCLC cells (Figure 4F). Next, we evaluated ferroptosis markers SLC7A11 and GPX4. Western blot and qRT-PCR results revealed that α-Hederin reduced the protein and mRNA levels of SLC7A11 and GPX4 in a dose-dependent manner (Figure 4G–4I). Ferroptosis inhibitor Ferrostatin-1 (Fer-1) was used to verify the mechanism of ferroptosis in α-Hederin against NSCLC cisplatin resistance. As expected, Fer-1 reversed the anti-proliferation and pro-apoptotic effects of α-Hederin in NSCLC cells (Supplementary Figure 1A, 1B). Furthermore, Fer-1 inhibited lipid peroxidation stimulated by α-Hederin in NSCLC cells (Supplementary Figure 1C, 1D). Next, as shown in Supplementary Figure 1E, Fer-1 reversed the anti-proliferation effects of α-Hederin in parental NSCLC cells and inhibited lipid peroxidation stimulated by α-Hederin in parental NSCLC cells (Supplementary Figure 1F, 1G). Which indicated α-Hederin-induced ferroptosis affected cisplatin-resistant cells which is a common phenotype in both cisplatin-resistant cells and parental cells.

### α-Hederin activated DDIT3/ATF3 pathway in NSCLC cells

**Figure 5:**
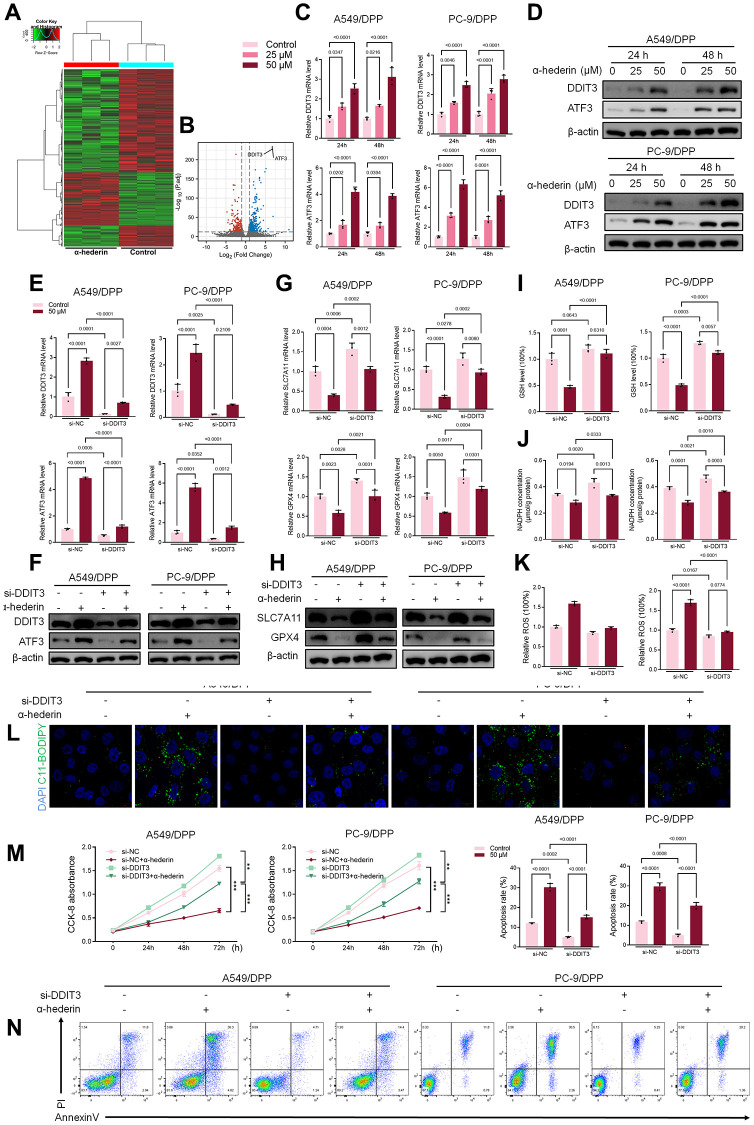
**α-Hederin activated DDIT3/ATF3 pathway in NSCLC cells.** (**A**) Heatmap of the inter-individual correlation of all mRNA transcripts (RNA-seq). (**B**) Volcano plot of gene expression. (**C**) qRT-PCR demonstrating DDIT3 and ATF3 mRNA levels in NSCLC cells after treatment with α-Hederin for 24 hours. (**D**) Western blot demonstrating DDIT3 and ATF3 protein levels in NSCLC cells after treatment with α-Hederin for 24 hours. (**E**, **F**) qRT-PCR and Western blot demonstrating DDIT3 and ATF3 levels in NSCLC cells after transfection with si-DDIT3 and treatment with α-Hederin for 24 hours. (**G**, **H**) qRT-PCR and Western blot demonstrating DDIT3, ATF3, SLC7A11 and GPX4 levels in NSCLC cells after transfection with si-DDIT3 and treatment with α-Hederin for 24 hours. (**I**–**K**) Cellular GSH, NADPH and ROS levels were detected in NSCLC cells after transfection with si-DDIT3 and treatment with α-Hederin for 24 hours. (**L**) Immunofluorescence assays were performed using C11-BODIPY (green) to determine oxidation and DAPI staining to detect the Nuclei (blue) in NSCLC cells. (**M**) CCK8 results showing the viability of NSCLC cells after transfection with si-DDIT3 and treatment with α-Hederin. (**N**) Flow cytometry detected NSCLC cells apoptosis after transfection with si-DDIT3 and treatment with α-Hederin for 24 hours. (*n* = 3). Data are shown as mean ± SD, One-way ANOVA, ^**^P < 0.01, ^***^P < 0.001.

Previous studies have reported that p53 represses SLC7A11 transcription and regulates cell ferroptosis [25]. PC-9 is a P53 mutant cell line [26]. Hence, we speculate that α-Hederin induced ferroptosis via a P53-independent pathway. RNA-seq was used to compare the mRNA differences between A549/DPP cells treated with or without 50 μM of α-Hederin for 24 hours. The results showed that DNA Damage Inducible Transcript 3 (DDIT3) and Activating Transcription Factor 3 (ATF3) were aberrantly expressed in cells treated with α-Hederin (Figure 5A, 5B). qRT-PCR and Western blot results were consistent with the RNA-seq results showing that α-Hederin activated the mRNA and protein levels of DDIT3 and ATF3 in a dose-dependent manner (Figure 5C, 5D). The TCGA database confirmed that the levels of DDIT3 and ATF3 in Lung squamous cell carcinoma (LUSC) and lung adenocarcinoma (LUAD) tumor tissues were significantly decreased compared with normal tissues. Besides, the ROC curves showed that DDIT3 and ATF3 had a promising ability to predict LUSC and LUAD in the TCGA cohort (Supplementary Figure 2A–2D). Furthermore, the Spearman rank correlation analysis showed a positive correlation between DDIT3 and ATF3 levels in LUSC and LUAD tumor tissues (Supplementary Figure 2E). Subsequently, A549/DPP and PC-9/DPP cell lines were transfected with DDIT3 small interfering RNA (si-DDIT3) for 24 hours. Figure 5E, 5F confirmed that si-DDIT3 significantly inhibited DITT3 and ATF3 levels in NSCLC cells. Previous research has reported that ATF3 regulates SLC7A11-mediated ferroptosis [27]. Hence, we assessed the role of DDIT3 in regulating ferroptosis. qRT-PCR and Western blot results showed that si-DDIT3 increased the mRNA and protein levels of ferroptosis markers SLC7A11 and GPX4 (Figure 5G, 5H). Downregulation of DDIT3 recovery cellular GSH and NADPH concentration and inhibited ROS level (Figure 5I–5K). Besides, Immunofluorescence using C11-BODIPY also showed a suppressive effect of si-DDIT3 on lipid peroxidation in NSCLC cells (Figure 5L). Furthermore, CCK8 and flow cytometry results suggested that si-DITT3 reversed the anti-proliferation and pro-apoptotic effect of α-Hederin in NSCLC cells (Figure 5M, 5N). Taken together, α-Hederin activated the DDIT3/ATF3 pathway and stimulated ferroptosis via inhibiting SLC7A11 and GPX4.

### miR-96-5p directly binds to DDIT3 in NSCLC cells

**Figure 6:**
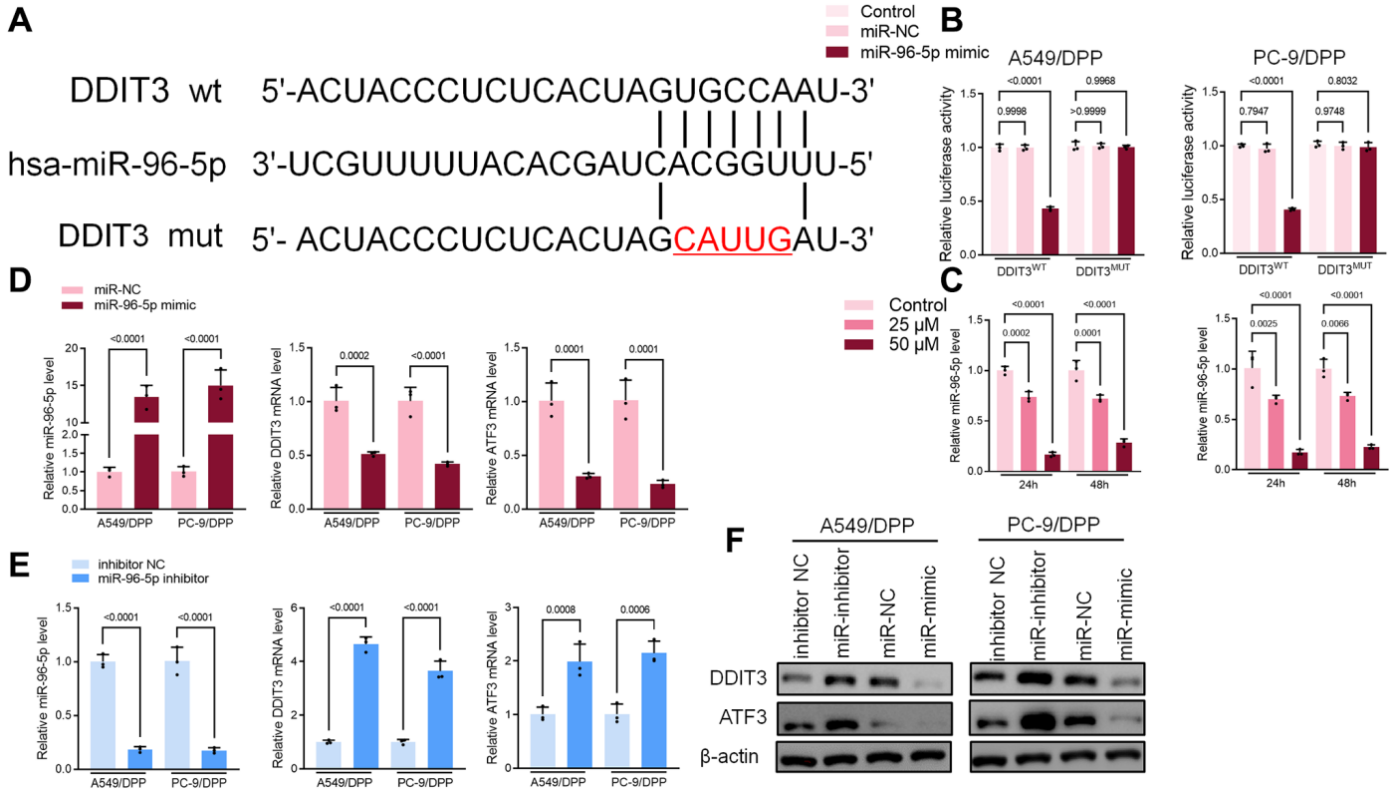
**miR-96-5p directly binds to DDIT3 in NSCLC cells.** (**A**) The predicted binding sites of miR-96-5p in DDIT3. The mutated (Mut) version of DDIT3 is also presented. (**B**) Relative luciferase activity in A549/DPP and PC-9/DPP cells was measured 48 h after transfection. (**C**) qRT-PCR demonstrating miR-96-5p levels in NSCLC cells after treatment with α-Hederin for 24 hours. (**D**, **E**) qRT-PCR demonstrating miR-96-5p, DDIT3, and ATF3 levels in NSCLC cells after transfection with miR-96-5p mimic or miR-96-5p inhibitor for 24 hours. (**F**) Western blot demonstrating DDIT3 and ATF3 levels in NSCLC cells after transfection with miR-96-5p mimic or miR-96-5p inhibitor for 24 hours. (*n* = 3). Data are shown as mean ± SD, One-way ANOVA.

Bioinformatics was used to predict the possible targets in determining the potential association between miR-96-5p and DDIT3. Results suggested that DDIT3 could be possible targets of miR-96-5p (Figure 6A). The dual-luciferase reporter assay results showed that overexpression of miR-96-5p reduced activity in NSCLC cells transfected with wild type (WT) DDIT3- vectors. However, no effect was seen in the cells transfected with the mutant type (MUT) DDIT3-vectors (Figure 6B). As shown in Figure 6A, the expression of miR-96-5p was blocked by α-Hederin treatment (Figure 6C). Besides, the TCGA database confirmed that the level of miR-96-5p in LUSC and LUAD tumor tissues was significantly increased compared with normal tissues (Supplementary Figure 2F, 2G). qRT-PCR and Western blot results showed that overexpression of miR-96-5p (miR-mimic) reduced the mRNA and protein levels of DDIT3. In contrast, inhibition of miR-96-5p (miR-inhibitor) increased the mRNA and protein levels of DDIT3 (Figure 6D, 6E).

### α-Hederin promoted EGR1 nuclear translocation and directly repressed miR-96-5p in NSCLC cells

**Figure 7:**
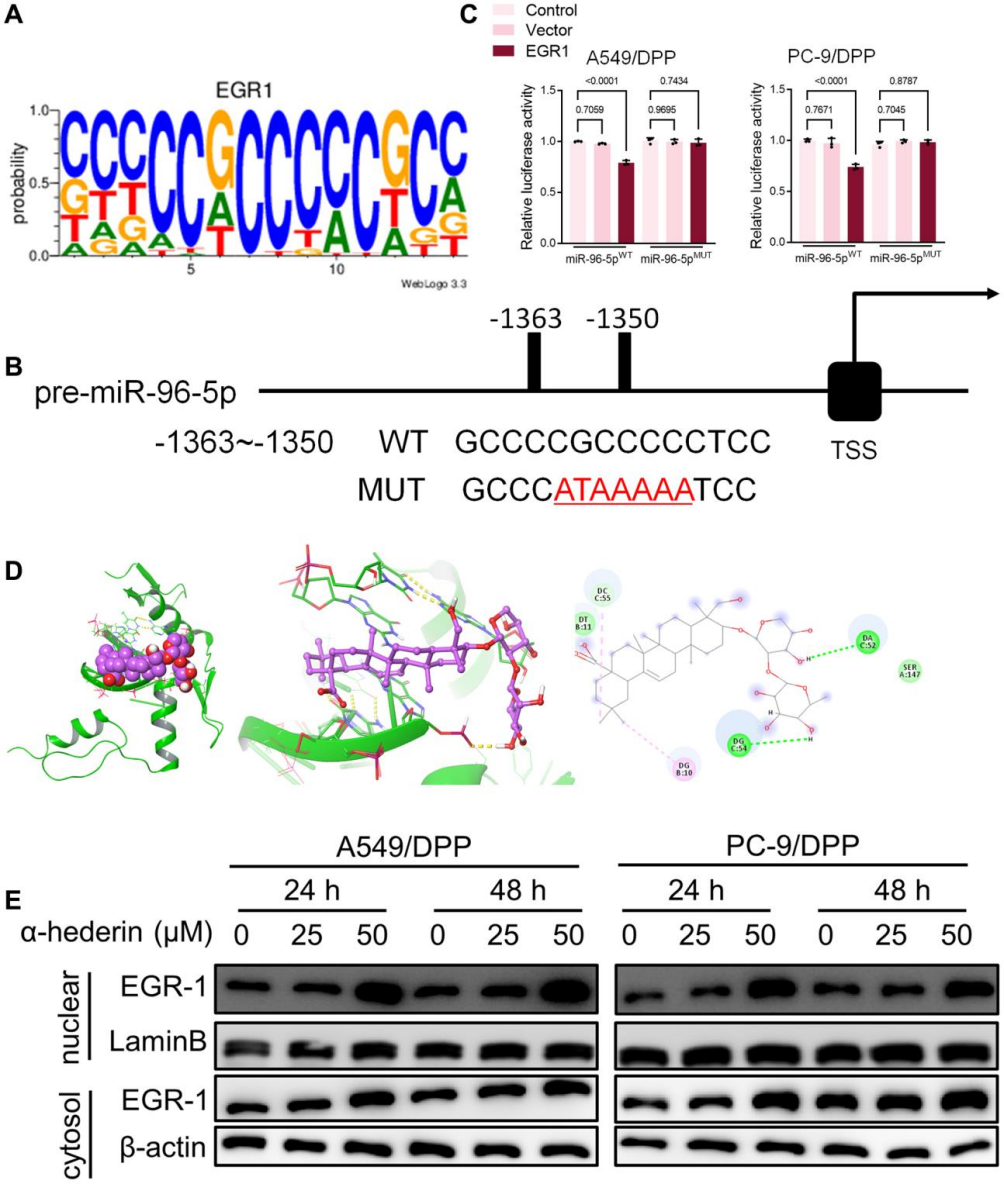
**EGR1 directly binds to the miR-96-5p promoter region.** (**A**) Sequence logo representing the consensus EGR1 binding motif from JASPAR database (http://jaspar.genereg.net/). (**B**) miR-96-5p promoter region was analyzed from the TSS. The mutation was seen from -1363~-1350. (**C**) The luciferase activity was detected in NSCLC cells after transfection with the EGR1-expressing vector. The luciferase reporter plasmid contained a 2 kb fragment upstream of the miR-96-5p stem-loop. (**D**) Theoretical binding mode of α-Hederin at the binding site of EGR1. (**E**) Western blot demonstrating EGR1 protein levels in nuclear and cytosol in NSCLC cells after treatment with α-Hederin for 24 hours. (*n* = 3). Data are shown as mean ± SD, One-way ANOVA.

**Figure 8:**
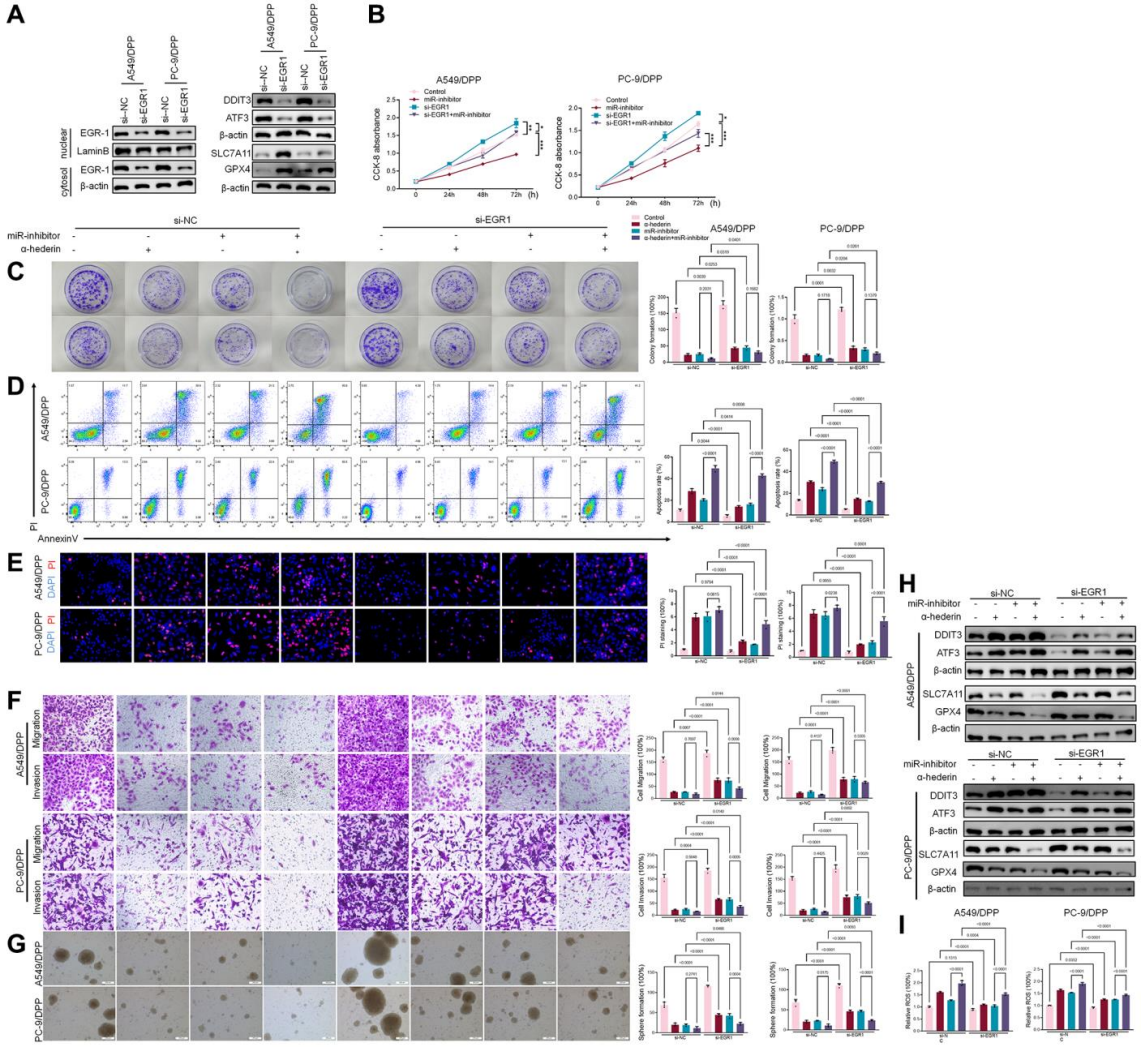
**α-Hederin promoted EGR1 nuclear translocation and directly repressed miR-96-5p in NSCLC.** (**A**) Western blot demonstrating EGR1 protein levels in nuclear and cytosol. DDIT3, ATF3, GPX4, and SLC7A11 protein levels are shown following transfection with si-EGR1 for 24 hours. (**B**) CCK8 results showing the viability of NSCLC cells after transfection with si-EGR1 or miR-96-5p mimic for 24 hours. (**C**) Colony formation assay showing proliferation in NSCLC cells after treatment with α-Hederin (50 μM) for 24 hours and transfection with si-EGR1 or miR-96-5p mimic for 24 hours. (**D**) Cell apoptosis was detected by flow cytometry. (**E**) Propidium iodide (PI) staining (red) indicates apoptotic/necrotic cells. (**F**) Cell migration and invasion were detected by Transwell assay. (**G**) Spheroid formation ability assessed in NSCLC cells. (**H**) Western blot demonstrating EGR1, DDIT3, ATF3, GPX4, and SLC7A11 protein levels in nuclear and cytosol of NSCLC cells. (**I**) Cellular ROS levels were detected in NSCLC cells after treatment with α-Hederin (50 μM) for 24 hours and transfection with si-EGR1 or miR-96-5p mimic for 24 hours. (*n* = 3). Data are shown as mean ± SD, One-way ANOVA, ^*^P < 0.05, ^**^P < 0.01, ^***^P < 0.001.

TransmiR v2.0 database (http://www.cuilab.cn/transmir/) was used to predict miR-96-5p associated transcription factor Early Growth Response 1 (EGR1). The EGR1 binding motif was predicted from JASPAR matrix models (Figure 7A). Next, we mutated the potential binding promoter site of miR-96-5p (−1363~−1350) (Figure 7B). An EGR1 expression vector containing a 3 kb fragment upstream of the miR-96-5p stem-loop was co-transfected into NSCLC cells. EGR1 significantly repressed the luciferase activity of miR-96-5p. However, miR-96-5p mutations showed no effect on EGR1 (Figure 7C). Molecular docking revealed that α-Hederin interacted with the active site of EGR1 (Figure 7D). Western blot results showed that α-Hederin significantly promoted EGR1 nuclear translocation in A549/DPP and PC-9/DPP cell lines (Figure 7E). Besides, the TCGA database confirmed that the level of EGR1 in LUSC and LUAD tumor tissues was significantly decreased compared with normal tissues (Supplementary Figure 2H, 2I). To elucidate the mechanism of EGR1 in NSCLC cells, we transfected EGR1 small interfering RNA (si-EGR1) into NSCLC cells. Figure 8A demonstrates that downregulation of EGR1 significantly suppressed the level of DDIT3 and ATF3 but enhanced SLC7A11 and GPX4. Further, we co-transfected the NSCLC cells with si-EGR1 and miR-96-5p inhibitors. The CCK-8 analysis results revealed that the downregulation of EGR1 increased cell viability. However, the miR-96-5p inhibitor abrogated this enhancement (Figure 8B). Furthermore, A549/DPP and PC-9/DPP cell lines were treated with 50 μM α-Hederin, co-transfected with si-EGR1 and miR-96-5p inhibitor, and cultured for 24 hours. As shown in Figure 8C–8H, downregulation of EGR1 reverses the effects of α-Hederin on cell proliferation, apoptosis, migration, invasion, and sphere-formation in A549/DPP and PC-9/DPP cell lines. At the same time, the inhibition of miR-96-5p abolished the effect of si-EGR1. Western blot assay results were consistent with these findings (Figure 8H). Besides, ROS results confirmed that overexpression of miR-96-5p or downregulation of EGR1 affects α-Hederin induced lipid peroxidation (Figure 8I). Therefore, these results demonstrate that α-Hederin promoted EGR1 nuclear translocation and directly repressed miR-96-5p in A549/DPP and PC-9/DPP cell lines.

## DISCUSSION

Currently, platinum-based chemotherapy remains the mainstay treatment in patients with advanced non-small-cell lung cancer without driver mutations. However, chemotherapy resistance in NSCLC greatly limits therapeutic effectiveness [7]. Ferroptosis is a novel form of cell death that was first defined in 2012. It is characterized by iron-dependent lipid peroxidation. Activation of lipid peroxidation depends on the inactivation of the GPX4 enzyme and the inhibition of GSH. GSH is a key molecule that inhibits cisplatin binding to DNA. Ferroptosis could be involved in regulating cisplatin resistance in tumor cells. Studies have demonstrated that artemisinin and its derivatives can effectively reverse cisplatin resistance in head and neck cancer, associated with ferroptosis induction [11]. Jin et al. showed that Solasonine, a natural compound, promoted ferroptosis of HCC cells *in vitro* and *in vivo* via GPX4 [28]. Besides, ferroptosis agonist erastin enhanced the sensitivity of NSCLC cells to CDDP via NRF2 [29]. This study found that α-Hederin inhibited GSH and activated lipid peroxidation in drug-resistant cell lines. Therefore, we hypothesized that induction of ferroptosis by α-Hederin plays a role in inhibiting NSCLC drug resistance.

DDIT3, a key transcription factor in the endoplasmic reticulum stress pathway, regulates apoptosis and inflammation [30]. Previous research showed that DDIT3 induced apoptosis by promoting the transcription of apoptosis-related molecules through the regulation of ATF family members such as ATF3 [31]. ATF3-mediated ferroptosis has been shown to play a key role in a variety of diseases such as gastric cancer [32], hepatocellular carcinoma [33], acute kidney injury [34], among others. Results of this study showed that α-Hederin activated the DDIT3/ATF3 pathway, thus promoting ferroptosis. In contrast, silencing DDIT3 suppressed ATF3 and abrogated ferroptosis in NSCLC cells. P53 mutations are present in 50% of all patients with non-small cell lung cancer and are the most common mutations associated with primary resistance to targeted therapy [35]. Hence, α-Hederin could activate ferroptosis through a P53-dependent pathway.

microRNAs (miRNAs) are small non-coding RNAs with approximately 18–22 nucleotides [36]. miRNAs have a key role in the post-transcriptional regulation of gene expression. By binding to specific sites in the 3′-UTRs of targeted mRNAs, miRNAs mediate their degradation and translational regulation. Deng et al. reported that miR-324 directly inhibited GPX4 and enhanced ferroptosis in cisplatin-resistant NSCLC cells. Besides, it has been demonstrated that miR-4443 repressed FSP1-mediated ferroptosis and promoted cisplatin resistance in NSCLC [37]. EGR1 is a member of the early growth response gene family whose expression is regulated by many extracellular signaling molecules and whose biological roles are closely related to cell proliferation, apoptosis, migration, invasion, and differentiation [38]. EGR1 expression was elevated in gastric cancer, prostate cancer, and glioblastoma compared to normal tissues. Increased EGRI expression significantly increased the proliferation, activity, and metastasis of cancer cells. High expression of EGR1 was also associated with poor prognosis in cancer patients [39–41].

EGR1 has a highly conserved DNA-binding domain, composed of three GysHis2 zinc finger junctions that specifically identify and bind the promoter regions of downstream genes, thereby acting as a transcriptional regulator [42]. EGR1 has been shown to target multiple miRNAs and influence the effects of these miRNAs on downstream target genes [43]. miR-152, an autophagy-regulated miRNA, was shown to be down-regulated in ovarian cancer resistant cells A2780/CP70. EGR1 has been shown to regulate the transcription of miR-152 by binding to the miR-152 promoter region. Up-regulation of both EGR1 and miR-152 inhibit the downstream target genes of miR-152, and ATG14, thus suppressing cisplatin resistance in ovarian cancer cells by preventing protective autophagy in ovarian cancer cells [44]. Our data revealed that α-Hederin promotes EGR1 translocation to the nucleus and directly binds to the miR-96-5p promoter region, thus suppressing miR-96-5p and sensitizing the NSCLC cells to cisplatin.

## CONCLUSION

This study demonstrated that α-Hederin was able to reverse cisplatin resistance in NSCLC *in vitro* and *in vivo*. This effect was mediated by activation of nuclear translocation of EGRI and direct repression of miR-96-5p which promoted DDIT3/ATF3-mediated ferroptosis.

## Supplementary Materials

Supplementary Materials and Methods

Supplementary Figures
