# Supplementary material for: α-Hederin promotes ferroptosis and reverses cisplatin chemoresistance in non-small cell lung cancer
Source: Aging (Albany NY). 2024 Jan 18;16(2):1298–317. doi: 10.18632/aging.205408 (PMC10866401; doi:10.18632/aging.205408)
Supplement: Supplementary Materials and Methods [file aging-16-205408-s001.pdf]

Supplementary Materials and Methods

Total RNA isolation and RT-qPCR assay

Total RNA was isolated using TRIzol (Invitrogen). Primers for EGR1, miR-96-5p, DDIT3, ATF3, SLC7A11 and GPX4 were attained from GenePharma (Shanghai, China). *β-actin* was used as the reference gene for mRNAs. U6 was used as an internal control for the level of miRNA expression. Gene expression was quantified using the $2^{-\Delta\Delta Ct}$ method as formerly described [1].

Protein extraction and Western blot analysis

Cytosolic and nuclear proteins in cells or tissues were isolated as described in NE-PER™ Nuclear and Cytoplasmic Extraction Reagents. and protein concentration was determined using a BCA protein assay kit. Approximately 30 μg of protein from each sample was separated by 10% sodium dodecyl sulphate-polyacrylamide gel electrophoresis (SDS-PAGE) and transferred to a polyvinylidene fluoride (PVDF) membrane. Membranes were blocked with 5% skimmed milk in TBST and incubated with primary antibodies overnight at 4°C [1]. Primary antibodies were obtained from Abcam (UK): CD44 (ab157107), CD133 (ab226355), E-Cadherin (ab40772), N-Cadherin (ab18203), Snail (ab216347), SLC7A11 (ab175186), GPX4 (ab231174), DDIT3 (ab11419), ATF3 (ab254268), EGR1 (ab194357), LaminB (ab16048) and β-actin (ab8226). All dilutions were 1:1,000 except β-actin is 1:3000.

Cell viability and clonability assays

According to the manufacturer's instructions, the cell counting kit-8 (CCK-8) system (Dojindo, Kumamoto, Japan) was used to measure the activity of transfected cells inoculated into 96-well plates at a density of 1×10^4 cells/wells. In short, before incubating the plate for 1 h at 37°C in the dark, 10 m of CCK-8 solution was added to each well, and measured the absorbance of each well at 450 nm with the microplate reader (Tecan, Männedorf, Switzerland). In the colony formation assay, cells were seeded with low density (1000 cells/plate) and cultured until visible clones appeared, then stained with Giemsa and counted the number of colonies.

Migration and invasion assays

In the Transwell migration assays and the invasion assays, the cells were first placed in serum-free medium, and the medium supplemented with 10% serum was placed in the lower chamber as a chemo-attractant. The former was seeded with 1×10^4 cells in an upper chamber with a non-coated membrane (24-well insert; 8 mm pore size; BD Biosciences, La Jolla, CA, USA), and the latter seeded with 2×10^5 cells with a Matrigel-coated membrane (24-well insert; 8 mm pore size; BD Biosciences). The cells were incubated in a tissue culture incubator at 37°C and 5% CO_2 for 16 h, after which the unmigrated/non-invasive cells on the upper sides of the Transwell membrane filter insert were gently wiped off with a cotton swab. On the underside of the insert, cells were stained with crystal violet and counted.

Apoptosis assay

Annexin V/PI staining was performed to assess cell apoptosis. In brief, cells (1×10^3) were seeded into the wells of 96-well plates and exposed to α-Hederin (50 μM) for one day, then trypsinized, washed twice with cold phosphate-buffered saline (PBS), resuspended in binding buffer (500 μL), and finally stained with annexin V-fluorescein isothiocyanate conjugate (5 μL) and PI (5 μL) at room temperature for 15 min in the dark. The proportion of apoptotic cells was determined by flow cytometry. Each experiment repeats three times.

Measurement of reactive oxygen species in cells

ROS in cells was measured using an H_2DCFDA probe as described previously [2]. To measure ROS levels in the liver, cold liver homogenate was centrifuged (10,000 g, 15 min, 4°C); supernatants were incubated with 10 μM H_2DCFDA in the dark for 1 h and subsequently transferred to a black-walled clear-bottomed 96-well plate. Fluorescence was immediately read at an excitation wavelength of 485–720 nm and an emission wavelength of 525–720 nm using a Synergy H4 spectrophotometer (BioTek, Winooski, VT, USA). Protein concentrations of the supernatants were quantified using BCA protein assay kits, calculated as units of fluorescence per microgram of protein, and concentrations presented as percentages of controls (% control).

Metabolomics data collection and analysis

A549/DPP cells treated with or without α-Hederin (50 μM) were used for metabolomics analysis. The cells were seeded into the wells of 6-well plates at an initial density of 106/well. After an appropriate culture period, the supernatant was discarded and each well was washed three times with PBS. Then, 2 mL of cold (4°C) methanol were added to each well and adhered cells were scraped free and lysed with a cell pulverizer to fully extract the metabolites. Finally, the supernatant was centrifuged at 14,000 × g for 10 min before liquid chromatography-mass spectro-

metry analysis. All resulting data were processed with Compound Discoverer 2.1 software (Thermo Fisher Scientific, Waltham, MA, USA). Orthogonal partial least squares discriminant analysis (OPLS-DA) and principal component analysis were conducted using SMICA-P 14.0 software (MKS Umetrics AB, Umeå, Sweden). Metabolite identification was based on product ion spectra and accurate masses. Pathway analysis was performed with MetaboAnalyst 4.0 metabolomics software.

High-throughput sequencing

For RNA sequencing (RNA-seq), libraries were generated from the total RNA by using TruSeq RNA Sample Preparation v2, according to the manufacturer's protocol. Samples were sequenced on the Illumina HiScanSQ platform (Illumina, San Diego, CA, USA). Reads were mapped to the human genome (Hg19) by using TopHat v2.0.6 (Johns-Hopkins University, Baltimore, MD, USA), and mRNA quanti-fication was performed by using Cuffdiff v2.0.2 (University of Maryland, College Park, MD, USA).

Luciferase reporter assay

For miRNA assay, the binding site of the 3′UTR of DDIT3, WT- DDIT3-3′UTR, and MUT-DDIT3-3′UTR were inserted into the KpnI and HindIII sites of the pGL3 promoter vector (HanBio, Shanghai, China) in a dual-luciferase reporter assay. Cells were plated into 24-well plates. Then, 80 ng plasmid, 5 ng Renilla luciferase vector pRL-SV40, 50 nM miR-96-5p mimics, and negative control were transfected into cells with Lipofectamine 2000 (Invitrogen). The cells were then collected and measured 48 h after transfection using a Dual-Luciferase Assay (Promega, Madison, WI, USA), following the manufacturer’s instructions.

For EGR1 assay, we constructed and inserted a 3 kb fragment upstream of the human miR-96-5p stem-loop with conserved Ebox motifs at -1350bp; then, constructed and inserted a 3 kb fragment upstream of miR-96-5p that contained mutations of E-Box motifs. Next, we inserted wild-type or mutated fragments into the luciferase reporter plasmid psiCHECK™-2 Vector (Sangon Biotech, Shanghai, China). Then, A549 and PC-9 cells were co-transfected with vectors over-expressing EGR1. Finally, the luciferase activity was observed.

Sphere formation assay

Cells were grown in MammoCult medium (Stem Cell Technologies, Vancouver, Canada) supplemented with MammoCult Proliferation Supplements (Stem Cell Technologies) and plated in 24-wells plates with ultra-low attachment at a density of 10,000 viable cells/mL and grown for 10 days. Spheres were counted and photographed.

Molecular docking analysis

Molecular docking analyses were performed to investigate interactions between α-Hederin and EGR1 using AutoDock Vina 1.1.2 [3]. A 3D structure of α-Hederin was drawn using ChemBioDraw Ultra 14.0 and converted to a 3D structure by ChemBio 3D Ultra 14.0. The 3D coordinates of EGR1 (PDB ID: 4X9J) were retrieved from the RCSB Protein Data Bank. The homology model was obtained from SWISS-MODEL (https://www.swissmodel.expasy.org/). AutoDockTools version 1.5.6 [3, 4] was employed to generate docking input files. The crystallographic ligands were extracted and fed into a docking database for redocking, and hydrogen atoms were added. An auxiliary program AutoGrid was used to generate a docking area that was defined as a 40 × 40 × 40 3D grid centered on the ligand binding site with a 0.375 Å grid space. All bond rotations for the ligands were ignored in this study. The best scoring pose from Vina docking score evaluations was selected for further analyses using PyMoL 1.7.6 software.

Animal studies

To examine the role of α-Hederin in a lung cancer metastasis model, the xenograft and the orthotopic experiments were performed with 5 mice in each group. We injected 1×10^6 A549/DPP intravenously through the tail vein into male nude mice (Chinese Science Academy, Shanghai, China). One month later, we measured and quantified the lung metastases by an *in vivo* bioluminescent imaging with an IVIS Lumina Series III *in vivo* Imaging System (PerkinElmer, New York, NY, USA). For one treatment cycle in a week (starting from week 1 to week 6), cisplatin (5 mg/kg, intraperitoneal injection, 1 time) and α-Hederin (40 mg/ kg, oral administration, 2 times) were given. Total three treatment cycles were conducted in this experiment.

As for xenograft assays, we injected 1×10^6 A549/DPP subcutaneously into the right side of each male nude mouse (n = 5) (Chinese Science Academy). We measured the tumor volumes (length × width^2 × 0.5) at specified time points. Tumors were excised 5 weeks after injection.

Histological detection

Tumor tissue was fixed in 4% paraformaldehyde and embedded in paraffin. Sections were stained with Ki67, E-cad, CD44 or Tunel to assess proliferation or apoptosis. Sections were detected via an Axiophot light

microscope (Zeiss, Jena, Germany) and captured with a digital camera.

Supplementary References

1. Zhang X, Hu F, Li C, Zheng X, Zhang B, Wang H, Tao G, Xu J, Zhang Y, Han B. OCT4&SOX2-specific cytotoxic T lymphocytes plus programmed cell death protein 1 inhibitor presented with synergistic effect on killing lung cancer stem-like cells in vitro and treating drug-resistant lung cancer mice in vivo. J Cell Physiol. 2019; 234:6758–68.
   https://doi.org/10.1002/jcp.27423
   PMID:30382588

2. Zhang J, Liang X, Li J, Yin H, Liu F, Hu C, Li L. Apigenin Attenuates Acetaminophen-Induced Hepatotoxicity by Activating AMP-Activated Protein Kinase/Carnitine Palmitoyltransferase I Pathway. Front Pharmacol. 2020; 11:549057.
   https://doi.org/10.3389/fphar.2020.549057
   PMID:33658919

3. Trott O, Olson AJ. AutoDock Vina: improving the speed and accuracy of docking with a new scoring function, efficient optimization, and multithreading. J Comput Chem. 2010; 31:455–61.
   https://doi.org/10.1002/jcc.21334
   PMID:19499576

4. Sanner MF. Python: a programming language for software integration and development. J Mol Graph Model. 1999; 17:57–61.
   PMID:10660911
