# Supplementary material for: α-Hederin promotes ferroptosis and reverses cisplatin chemoresistance in non-small cell lung cancer
Source: Aging (Albany NY). 2024 Jan 18;16(2):1298–317. doi: 10.18632/aging.205408 (PMC10866401; doi:10.18632/aging.205408)
Supplement: Supplementary Figures [file aging-16-205408-s002.pdf]

SUPPLEMENTARY FIGURES

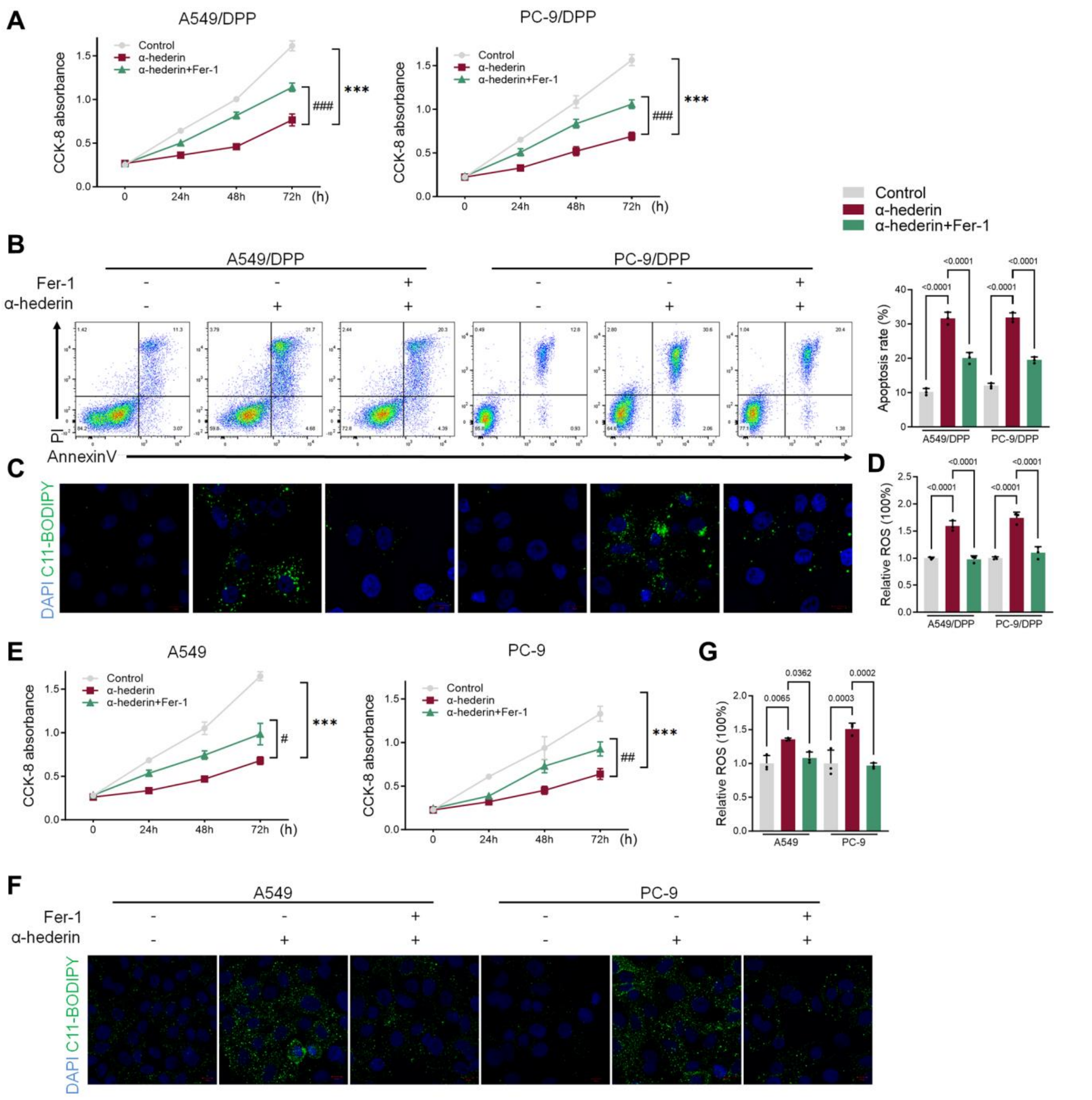

Supplementary Figure 1. Inhibition of ferroptosis reversed the effect of α-Hederin. (**A**) CCK8 results showing the viability of A549/DPP and PC-9/DPP cells after treatment with α-Hederin (50 μM) and Fer-1 (5 μM) for 24 hours. (**B**) Cell apoptosis was detected by flow cytometry. (**C**) Immunofluorescence assays were performed using C11-BODIPY (green) to determine oxidation and DAPI staining (blue) to detect Nuclei in A549/DPP and PC-9/DPP cells. (**D**) Cellular ROS levels were detected in A549/DPP and PC-9/DPP cells after treatment with α-Hederin (50 μM) and Fer-1 (5 μM) for 24 hours. (**E**) CCK8 results showing the viability of A549 and PC-9 cells after treatment with α-Hederin (50 μM) and Fer-1 (5 μM) for 24 hours. (**F**) Immunofluorescence assays were performed using C11-BODIPY (green) to determine oxidation and DAPI staining (blue) to detect Nuclei in A549 and PC-9cells. (**G**) Cellular ROS levels were detected in A549 and PC-9 cells after treatment with α-Hederin (50 μM) and Fer-1 (5 μM) for 24 hours. (*n* = 3). Data are shown as mean ± SD, One-way ANOVA.

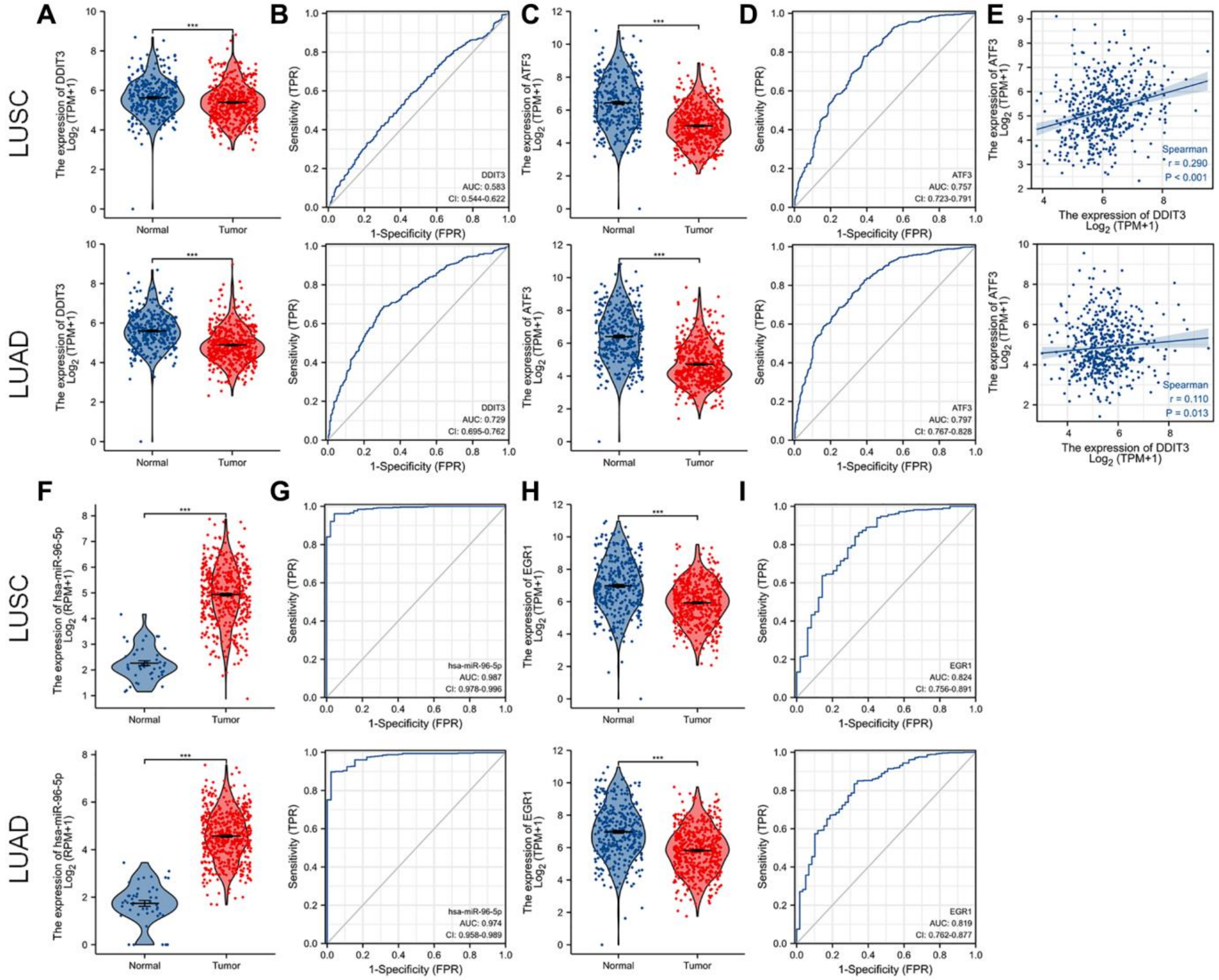

Supplementary Figure 2. TCGA analysis of DDIT3, ATF3 and EGR1. (**A**) DDIT3 levels in LUSC and LUAD tumor tissues in the TCGA database. (**B**) ROC curves for DDIT3 expression in LUSC and LUAD patients in the TCGA database. (**C**) ATF3 levels in LUSC and LUAD tumor tissues in the TCGA database. (**D**) ROC curves for ATF3 expression in LUSC and LUAD patients in the TCGA database. (**E**) Spearman correlation analysis was used to determine the correlation between DDIT3 and ATF3 in LUSC and LUAD in the TCGA database. (**F**) miR-96-5p levels in LUSC and LUAD tumor tissues in the TCGA database. (**G**) ROC curves for miR-96-5p expression in LUSC and LUAD in the TCGA database. (**H**) EGR1 levels in LUSC and LUAD tumor tissues in the TCGA database. (**I**) ROC curves for EGR1 expression in LUSC and LUAD in the TCGA database. Data are shown as mean ± SD, ***One way ANOVA $P < 0.001$.
